# Recent advances in autoimmune encephalitis

**DOI:** 10.1055/s-0044-1793933

**Published:** 2024-12-20

**Authors:** João Henrique Fregadolli Ferreira, Caio César Diniz Disserol, Bruna de Freitas Dias, Alexandre Coelho Marques, Marina Driemeier Cardoso, Pedro Victor de Castro Silva, Fabio Fieni Toso, Lívia Almeida Dutra

**Affiliations:** 1 Hospital Israelita Albert Einstein, Instituto do Cérebro, São Paulo SP, Brazil.; 2 Universidade Federal do Paraná, Hospital de Clínicas, Curitiba PR, Brazil.; 3 Instituto de Neurologia de Curitiba, Curitiba PR, Brazil.

**Keywords:** Autoimmune Diseases of the Nervous System, Anti-N-Methyl-D-Aspartate Receptor Encephalitis, Rituximab, Doenças Autoimunes do Sistema Nervoso, Encefalite Antirreceptor de N-Metil-D-Aspartato, Rituximab

## Abstract

Since the description of autoimmune encephalitis (AE) associated with N-methyl-D-aspartate receptor antibodies (anti-NMDARE) in 2007, more than 12 other clinical syndromes and antibodies have been reported. In this article, we review recent advances in pathophysiology, genetics, diagnosis pitfalls, and clinical phenotypes of AE associated with cell surface antibodies and anti-GAD associated neurological syndromes. Genetic studies reported human leukocyte antigen (HLA) associations for anti-LGI1, anti-Caspr2, anti-IgLON5, and anti-GAD. Follow-up studies characterized cognitive dysfunction, psychiatric symptoms, sleep disorders, and adaptative behavior dysfunction, mainly for anti-NMDARE. Late-onset anti-NMDARE and anti- GABA-B receptor (GABA-BR) encephalitis patients were described to have worse prognoses and different tumor associations. Additionally, the clinical spectrum of anti-LGI1, anti-AMPAR, anti-CASPR2, and anti-IgLON5 was expanded, comprising new differential diagnoses. The diagnostic criteria for AE were adapted to the pediatric population, and a diagnostic algorithm was proposed, considering potential mimics and misdiagnosis. We also review the limitations of commercial assays for AE and treatment recommendations, as well as clinical scales for short and long-term assessment of AE patients, along with cognitive evaluation.

## Resumo

Desde a descrição da encefalite autoimune (EA) associada a anticorpos contra o receptor N-methyl-D-aspartate (anti-NMDARE) em 2007, mais de 12 síndromes clínicas e anticorpos foram reportados. Neste artigo, revisamos avanços recentes na fisiopatologia, genética, diagnóstico e fenótipos clínicos da EA associada a anticorpos contra antígenos de superfície e das síndromes neurológicas associadas aos anticorpos anti-acido glutâmico decarboxilase ( *glutamic acid decarboxylase* , GAD, em inglês). Estudos genéticos revelaram associações do antígeno leucocitário humano ( *human leukocyte antigen* , HLA, en inglês) com as EAs anti-LGI1, anti-Caspr2, anti-IgLON5 e anti-GAD. Estudos de seguimento caracterizaram disfunção cognitiva, sintomas psiquiátricos, distúrbios do sono e disfunção do comportamento adaptativo, principalmente para anti-NMDARE. Apresentações tardias de anti-NMDARE e anti-GABA-BR foram associadas a outros tumores e a pior desfecho. Ademais, o fenótipo clínico de anti-LGI1, anti-AMPAR, anti-CASPR2 e anti-IgLON5 foi expandido, englobando outros diagnósticos diferenciais. Os critérios diagnósticos para a EA foram adaptados para a população pediátrica, e foi proposto um novo algoritmo diagnóstico levando em consideração potenciais condições mimetizadoras e erros diagnósticos. Foram revisadas também as limitações dos kits comerciais para testagem, recomendações atuais para o tratamento e escalas clínicas para o seguimento de curto e longo-prazo dos pacientes, incluindo a avaliação cognitiva.

## INTRODUCTION

Autoimmune encephalitis (AE) is a group of inflammatory disorders caused by autoantibodies targeting neuronal cell surface or synaptic proteins, leading to neuronal dysfunction. 1 It is a rare disease, with an annual incidence estimated at 1.2 cases/100 thousand inhabitants, 2 and a costly condition that often requires intensive care unit (ICU) care, prolonged hospital stays, and immunosuppressant treatments. 3 4 Autoimmune encephalitis represents a significant social and financial burden as it often affects young patients who may not return to previous daily activities. 5 6

Since the description of anti-N-methyl-D-aspartate receptor encephalitis (anti-NMDARE) in 2007, 7 more than 12 other clinical syndromes and antibodies have been reported. 1 New insights into pathophysiology indicate potential treatment targets for refractory patients, and genetics studies evaluated predisposition to AE. Recent data shed light on the accuracy of available diagnostic methods and the sensitivity of the clinical criteria, emphasizing strategies to avoid misdiagnosis. 8 9 10 Also, follow-up studies characterized cognitive, behavioral, and psychiatric outcomes.

The present review addresses AE associated with antibodies (ABs) against cell surface antigens and anti-GAD65-associated neurological syndromes. Because paraneoplastic neurologic syndromes associated with high-risk ABs (previously known as onconeural ABs) have different pathophysiology, prognosis, and response to treatment, 11 they will not be addressed.

## CLINICAL SYNDROMES AND PATHOPHYSIOLOGY

**Figure 1:**
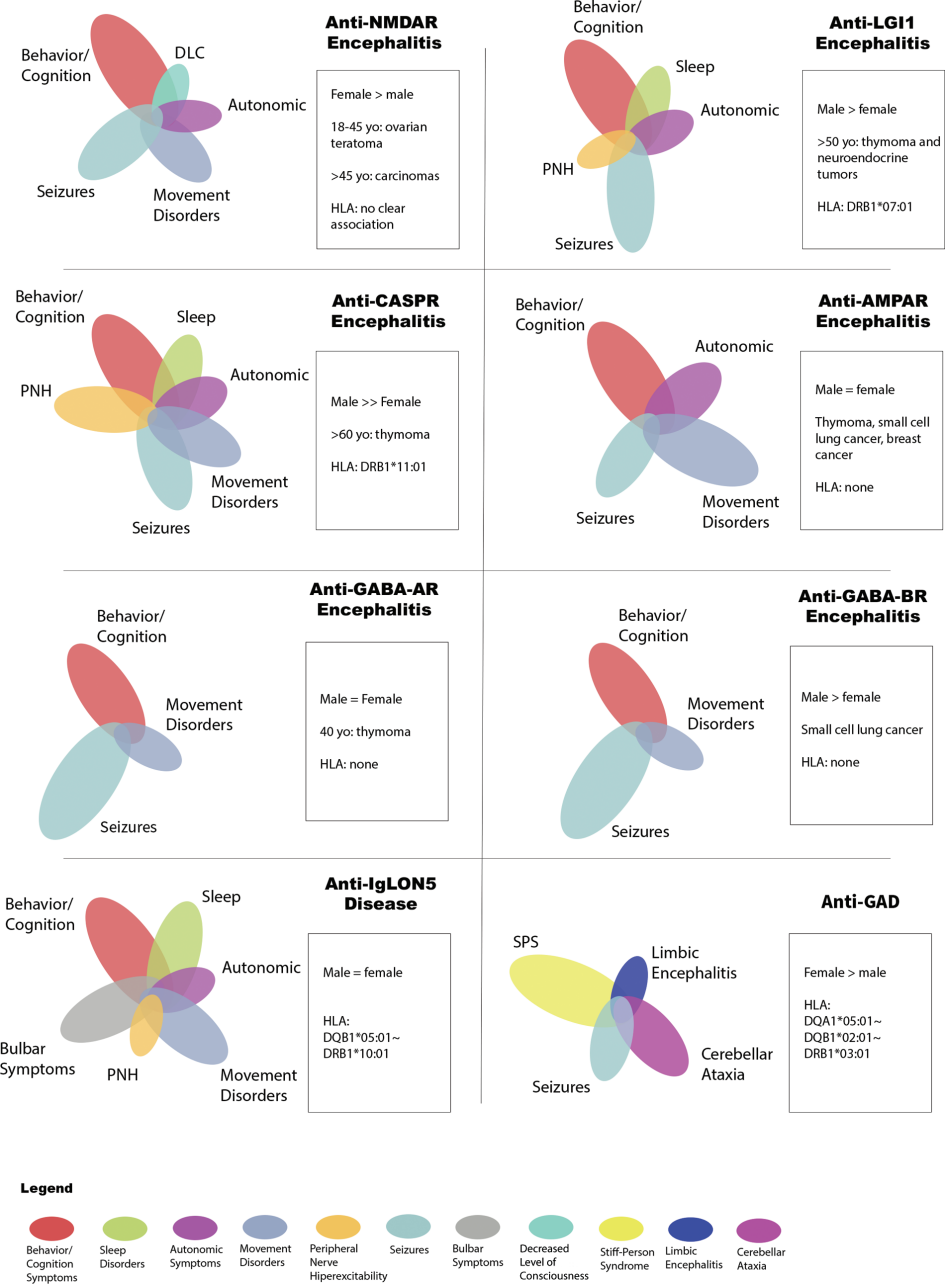
Clinical spectrum of autoimmune encephalitis. Observe the different clinical phenotypes of each antibody reported.

Overall, AE manifests with rapidly progressive (≤ 12 weeks) neurological symptoms, such as psychiatric symptoms, memory and cognitive deficits, seizures, movement disorders, dysautonomia, and decreased level of consciousness. 12 However, the clinical spectrum of many AE subtypes continues to expand ( Figure 1 ).

## Anti-NMDARE

Anti-N-methyl-D-aspartate receptor encephalitis is caused by ABs targeting the GluN1 subunit of the NMDA receptor, which causes cross-linking and internalization of those receptors, leading to impaired NMDAR electric currents. 13 14 Animal models showed that anti-NMDAR ABs modify CA1 pyramidal cells' excitability and hippocampal network activity. 15 Additionally, hippocampal proteomics revealed changes in components of glutamatergic, GABAergic, and central hubs of intracellular signaling, providing insights into molecular mechanisms for electrophysiological findings and pathophysiology. 15

Recently, the mean annualized incidence rate of anti-NMDARE was estimated at 1.00/million cases in the Netherlands. 8 Anti-NMDARE predominates in young women, with an ovarian teratoma found in nearly 50% of cases. 16 However, reports from China, Australia, and Brazil showed that up to 40% of the patients were male, and lower rates of neoplasia were found (10%), suggesting that genetic and environmental factors may play a role in the development of the disease. 17 18 19 Moreover, one recent case series showed that 20% of patients were > 45 years, indicating that late presentation may be more common than previously expected. 10 These late-onset cases were associated with other tumors, mainly carcinomas, were oligosymptomatic, and had worse outcome. 10 16

The classic anti-NMDARE clinical syndrome starts with prodromal symptoms, followed by cognitive or psychiatric manifestations (delusions, psychosis, catatonia), movement disorders (orofacial dyskinesias, choreoathetosis), speech disorder, dysautonomia, seizures, and decreased level of consciousness. 12 16

A substantial number of patients require ICU admission for management of status epilepticus, autonomic dysfunction, and mechanical ventilation. 4 Clinical variables from the acute presentation can be used to evaluate disease prognosis. The anti-NMDAR Encephalitis One-Year Functional Status (NEOS) score predicts functional outcomes after 1 year of symptoms onset and includes 5 clinical variables:

- ICU admission required;
- lack of clinical improvement after 4 weeks of treatment;
- lack of treatment within 4 weeks of symptoms onset;
- abnormal magnetic resonance imaging [MRI);
- pleocytosis (white blood cell [WBC] count > 20 cells/mm ^3^ ). 20

This score also performed well for children, and preliminary findings indicate that it can also be used for cognitive outcomes. 21

In the post-acute phase, patients may present a neuropsychiatric syndrome that resembles schizophrenia, which improves gradually. 22 Sleep impairment is also prevalent during the recovery phase, mainly hypersomnia and confusional arousal, along with behavioral symptoms (hyperphagia, hypersexuality), 23 mood disorders, 22 24 and adaptative behavior dysfunction. 25 These symptoms can be debilitating and should be actively screened during follow-up interviews.

In the chronic phase of the disease, cognitive decline is prevalent. 5 6 After a median follow-up of 4.9 years, two-thirds of patients may still present moderate-to-severe cognitive deficits, indicating that the patient's recovery may take several years. 6 In children, school difficulties are reported in 36 to 70% after a follow-up of 2.5 to 3 years, 5 26 and even after a median follow-up of 7 years, 27% remain sociofamiliar dependent. 27 Additionally, younger patients (< 6 years) were reported to present more impairment in adaptative behavior 25 and worse outcomes using the Liverpool Outcome Score (LOS), a multidomain tool that evaluates physical, cognitive, and psychological variables. 27

Approximately 25% of patients present relapses, and these episodes are often less severe than the initial event. 28 29 Differentiating disease activity and relapses from neurological sequelae is a major challenge in clinical practice, and future studies are needed to address this question.

## Anti-LGI1 encephalitis

Leucine-rich-glioma-inactivated 1 (LGI1) is a synaptic protein associated with the formation of a transsynaptic linker molecule in excitatory synapses. 30 31 After binding with proteins ADAM23 and ADAM22, LGI1regulates the presynaptic voltage-gated potassium channel (VGKC) and postsynaptic AMPA receptor. 31 The binding of anti-LGI1 antibodies alters the expression and clustering of those ion channels and results in neuronal hyperexcitability. 30

Up to 90% of patients with anti-LGI1 encephalitis present with limbic encephalitis (LE) at some point in the disease, along with seizures and cognitive decline. 24 32 33 Faciobrachial dystonic seizures (FBDSs) are considered highly specific and frequently precede encephalitis, representing a window of opportunity for early diagnosis and treatment. 34 Multifocal seizures, including autonomic and pilomotor seizures, and hyponatremia are also tips for diagnosis. 35

In the post-acute phase, patients frequently present subclinical focal seizures, FBDSs, and sleep disturbances (insomnia, REM-sleep behavior disorder, wake after sleep onset, Morvan-syndrome-like manipulatory behaviors), which could impair cognitive recovery and contribute to depressive symptoms. These residual symptoms are responsive to treatment and should be actively screened with electroencephalogram (EEG) and polysomnography (PSG). 36

Follow-up studies revealed that only about ⅓ of patients actually return to previous daily activities, 24 32 and despite recovery of functional independence, most patients remain with cognitive impairment. 37 38 Additionally, mesial temporal sclerosis (44%) and hippocampal atrophy (40–95%) were reported, even in patients with normal brain MRI during the acute phase. 32 38

## Anti-CASPR2 encephalitis

Contactin-associated protein 2 (Caspr2) is an adhesion protein localized in the juxta paranodal region that binds to contactin-2. Anti-Caspr2 ABs inhibit that interaction, impairing VGKC clustering and causing hyperexcitability through unknown mechanisms. 39

Anti-Caspr2 encephalitis is more common in males > 50 years (75–90%). Limbic encephalitis is the most common clinical phenotype, frequently overlapping with acquired neuromyotonia or Morvan syndrome. 40 41 Seizures, dysautonomia, ataxia, and neuropathic pain are also reported as core symptoms, often associated with nonspecific manifestations (insomnia, asthenia, weight loss, and/or mood disorders). 42 Diagnosis may be challenging as core symptoms are rarely present at onset, and disease progression is slow, with the disease peak potentially taking over a year. 42

Tremor, episodic ataxia, paroxysmal orthostatic segmental myoclonus of the lower limbs, and continuous segmental spinal myoclonus were reported as initial symptoms or the sole manifestation of the disease. 43 Follow-up studies revealed a good functional prognosis for most patients (modified Rankin Scale [mRS] scale ≤ 2), although with high mortality (up to 17%), probably related to the older age and severity of this subtype of encephalitis. 41

## Anti-AMPAR encephalitis

The α-amino-3-hydroxy-5-methyl-4-isoxazolepropionic acid receptor (AMPAR) is a glutamate inotropic receptor present in excitatory central nervous system synapses. AMPAR Abs may alter the receptor distribution, impair synaptic plasticity, and cause symptoms in mice. 44 45 The clinical syndrome is characterized mostly by LE (cognitive impairment, behavioral/psychiatric symptoms, and seizures) or symptoms of limbic dysfunction associated with more diffuse signs of inflammation on brain MRI. 46 Autonomic dysfunction, movement disorders, and cerebellar ataxia were also reported 47 ( Figure 1 ).

Three patients with acute/subacute global amnestic syndrome as sole manifestation were described, which lasted for several weeks, and one patient presented normal paraclinical investigation (MRI and CSF). In the acute phase, those cases can be difficult to differentiate from other amnestic syndromes, such as transient global amnesia (TGA), dissociative amnesia, or epileptic syndromes. 48

## Anti-GABA-AR encephalitis

Gamma-aminobutyric acid-A receptors (GABA-AR) are ion-selective channels that are associated with neuronal excitability inhibition. Anti-GABA-AR Abs induce conformational changes and antagonism of inhibitory neurotransmission. 49

Anti-GABA-AR encephalitis affects a broad age range (2.5 months–88 years) and both genders. 50 Seizures are the most common clinical manifestation, including refractory status epilepticus and epilepsia partialis continua. 51 52 Cognitive impairment, behavioral symptoms, movement disorders (ataxia, choreoathetosis), and decreased level of consciousness are also core symptoms. 50 Brain MRI shows distinctive lesions, characterized by multifocal cortico-subcortical T2/FLAIR lesions, present in 77% of patients. 50 53 Normal brain MRI appears to be rare (11%, 3/26) in this AE subtype. 50

## Anti-GABA-BR encephalitis

Gamma-aminobutyric acid-B receptors (GABA-BR) are G-protein-coupled, and their function is associated with neuronal inhibitory activity. 54 The most frequent anti-GABA-BR encephalitis clinical syndrome is characterized by prominent seizures and LE. Rarely, cerebellar ataxia and opsoclonus myoclonus syndrome are present. 55 Patients with rapidly progressive dementia, myoclonus, and cerebellar/pyramidal findings were also reported, raising suspicion for Creutzfeldt-Jakob disease (CJD). 56

There is a strong association with tumors (over 60%), mainly SCLC, and older patients carry the highest risk. 57 58 Other neoplasia reported include pulmonary epithelioid hemangioendothelioma, esophageal cancer, and laryngeal cancer. 58 Co-occurrence of paraneoplastic antibodies is frequent in anti-GABA-BR encephalitis, with anti-Hu and ani-SOX1 being the most common. Antibodies against GABA-BR accessory protein KCTD16 were identified as a biomarker for SCLC 56 and should be requested to all patients without a known tumor, to help with screening guidance. 57 Two recent Asians series found a lower frequency of tumors (33.9%), suggesting the presence of genetic and environmental factors in the disease pathophysiology. 58 59

## Anti-IGLON5 antibody-associated disease

Anti-IgLON5 Abs are considered pathogenic, 60 nonetheless, disease pathophysiology remains unknown. 61 Although preliminary neuropathological studies showed abnormal aggregates of hyperphosphorylated tau on the brainstem tegmentum and hypothalamus, 62 63 a recent autopsy cohort described two patients without brainstem taupathy. 64 Moreover, despite initial data showing a strong association with human leukocyte antigen (HLA) DRB1*10 and 01-DQB1*05:01, the disease can occur without the classical HLA association. 65

The clinical phenotype of anti-IgLON5 disease was initially described as bulbar dysfunction, sleep disorders (stridor, obstructive sleep apnea, REM and NREM parasomnias), abnormal movements (myoclonus, chorea, parkinsonism), gait instability, and cognitive decline, 62 66 and it continues to expand. Peripheral nervous system (PNS) involvement can be present (neuromyotonia, fasciculations), 67 and a motor neuron disease-like (MND-like) phenotype has been described. 68 69 A new clinical score was developed to assess the severity and progression of core clinical symptoms. 70

Symptoms can be slowly progressive, and there may be difficulties in differentiating them from those of neurodegenerative diseases. Patients with MND associated with vocal cord paralysis, parasomnias, or involuntary movements should be tested for anti-IgLON5 ABs. 68 Other differential diagnoses include Huntington's disease, progressive supranuclear paralysis (PSP), and multiple system atrophy. 66 71

## Anti-GAD-associated neurological syndromes

Anti-GAD ABs lead to reduced synaptic GABA and, thus, enhance glutamatergic activity. 72 Direct pathogenicity of anti-GAD ABs has not been demonstrated, but high tilters (enzyme-linked immunosorbent assay [ELISA] 10,000 IU/mL in serum and 100 IU/mL in CSF) are associated with specific neurologic phenotypes: stiff person syndrome (SPS), LE, autoimmune epilepsy, cerebellar ataxia (CA), or overlap syndromes. 73 Patients with lower titers of anti-GAD ABs have nonspecific syndromes, and very low titers are seen in diabetes patients or have unclear significance. 73 Patients with high serum titers (> 10,000 IU/mL) also have anti-GAD detected in the cerebrospinal fluid (CSF). 74 Thus, CSF analysis should be performed in patients with clinically suspicious but low serum titers of anti-GAD. In patients without a classic neurological phenotype but with anti-GAD ABs detected in the serum and CSF, the intrathecal synthesis of GAD Abs, can also be used as an indicator that a neurologic syndrome is associated with GAD autoimmunity. 72

## DIAGNOSTIC CRITERIA AND AE MIMICS

Graus' classical diagnostic criteria for AE were validated for the Chinese, 75 Dutch, 8 and American 76 adult populations. The sensitivity of the possible AE criteria ranged from 83 to 84%, while specificity ranged from 27 to 94%, 8 75 and the positive predictive value was 47%. 8 Those findings indicate that some patients may not fulfill the classical criteria, and this topic is still a matter of debate in the literature, suggesting that progressive and atypical forms may occur. Preliminary data showed that those patients with AE not fulfilling the criteria have chronic and/or oligosymptomatic onset, mainly associated with anti-GAD65, anti-LGi1, anti-Caspr2, and anti-IgLON5. 10 Also, one retrospective study revealed that 0.8% of patients with a presumed diagnosis of neurodegenerative dementias may have AE. 77 Those AE patients did not fulfill the rapidly progressive dementia criteria, but presented atypical findings for neurodegenerative diseases (subacute worsening at some point of the disease, myoclonus, history of other autoimmune disease, fluctuating course, and seizures). Thus, although rare, some patients with atypical clinical presentations do not fulfill the AE clinical criteria, and findings considered suspicious of an antibody-mediated clinical manifestation should be considered for testing.

Because of the unique aspects of the developing brain and difficulties in evaluating memory and behavior in children, the AE criteria for the pediatric population were developed, highlighting the importance of paraclinical findings of inflammation for the diagnosis. 78 The pediatric criteria suggest that AE should be considered mainly in previously healthy children and propose other differential diagnoses, such as genetic diseases.

**Figure 2:**
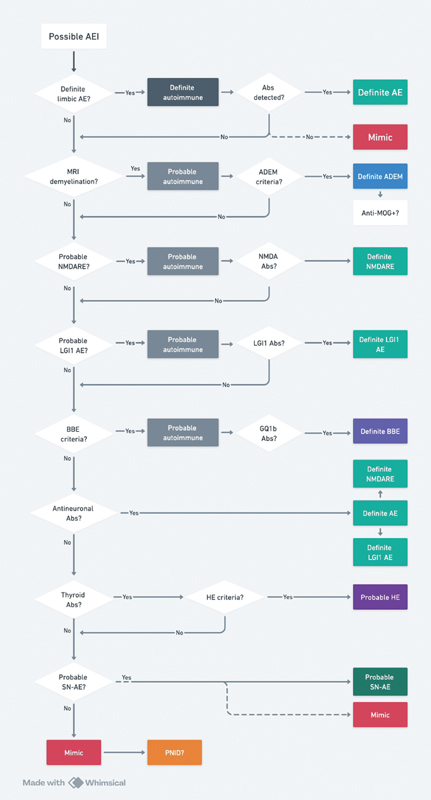
Abbreviations: ABs, antibodies; ADEM, acute disseminated encephalomyelitis; AE, autoimmune encephalitis; BBE, Bickerstaff brainstem encephalitis; HE, Hashimoto encephalopathy; MOG, myelin oligodendrocyte glycoprotein; MRI, magnetic resonance imaging; NMDARE, anti-NMDA receptor encephalitis; PNID, probable neuroinflammatory disorder; SN-AE, seronegative autoimmune encephalitis. Diagnostic algorithm for autoimmune encephalitis (adapted from Van Steenhoven et al. 8 ). Begin the algorithm from the entry criteria of possible AE, then apply the subsequent criteria (MRI demyelination, probable anti-NMDARE, probable anti-LGI1 encephalitis, BBE). Patients with positive Abs are classified as definite AE. In case of negative testing, evaluate thyroid ABs, probable SN-AE criteria, AE mimics (including PNID).

An adaptation of the AE diagnostic criteria algorithm was proposed to incorporate specific information for anti-NMDARE and anti-LGI1 encephalitis and to prevent misdiagnosis ( Figure 2 ). 8 In this adaptation, possible AE is the entry criteria, followed by the sequential application of classical AE syndromes and acute disseminated encephalomyelitis (ADEM) criteria. Novelties included the criteria for probable neuroinflammatory disorder (PNID) and AE mimics. 8

Classification as probable anti-LGI1 encephalitis relies on the presence of subacute onset (< 3 months) cognitive dysfunction associated with faciobrachial dystonic seizures or frequent (> 5 per day) stereotypical focal seizures (excluded alternative diagnoses). The sensitivity of anti-LGI1 encephalitis criteria was 66%, and specificity was 96%, that may lead to earlier treatment in some patients. 8 The sensitivity of the clinical criteria for anti-NMDARE is 49 to 50%, and specificity is 96 to 98%. 75 79 Definite criteria for LE require clinical and paraclinical tests only, and despite its low sensitivity (10–38%), it is highly specific (96–98%). Bickerstaff brainstem encephalitis (BBE) is included under the umbrella of anti-GQ1b antibody syndromes, and antibody detection is higher in the first week of symptoms, with a sensitivity of 74% with ELISA or glycoarray (and 82% if both are combined). 80

PNID is a new concept introduced to comprise inflammatory disorders that require immunotherapy but do not fulfill any formal AE criteria. It is defined by 2 or more of the following: brain MRI suggestive of AE, CSF pleocytosis, CSF-specific oligoclonal band, repeated steroid responsiveness, or similar staining pattern on immunohistochemistry (IHC) in serum, and CSF in the absence of a known neuronal autoantibody. Caution is needed over PNID due to high etiological heterogeneity. For these reasons, PNID is considered a subtype of AE mimic. 8

The term probable antibody-negative AE should be used for the patients with negative results that fulfill the clinically available criteria, which require two of the following:

- brain MRI suggestive of inflammation;
- pleocytosis or presence of oligoclonal bands;
- brain biopsy showing inflammatory infiltrate. 12

Observe that CSF protein level is not a criterion. Moreover, studies showed that many patients considered to have antibody-negative AE were not adequately screened with laboratory techniques for antineuronal ABs detection in serum and CSF, which may lead to misdiagnosis. If the patient does not fulfill the criteria for probable antibody-negative AE, an alternate diagnosis should be investigated.

Autoimmune encephalitis mimics are conditions that can present with similar clinical features to AE but are distinct entities. Common AE mimics are primary psychiatric and functional neurological disorders, neurodegenerative and genetic diseases, epilepsy, neoplasms, and infections, among others. 8 9 81 82 Because the yield of testing is very low in patients with isolated first-episode psychosis, patients should not be routinely tested. 83 84 One series revealed that most misdiagnosed patients (72%) did not fulfill the possible AE criteria, 9 highlighting the importance of adhesion to the established clinical criteria.

## NEURONAL ANTIBODIES TESTING

Antineuronal AB detection requires two complementary techniques, namely tissue-based immunofluorescence assays (TBAs) and cell-based assays (CBAs); CSF and serum should be screened simultaneously to enhance diagnostic accuracy, due to variations in sensitivity depending on the samples analyzed. 31 85 For instance, anti-NMDAR CBA sensitivity is 68% in serum and 99% in CSF. 10 Overinterpretation of isolated positive CBA results in serum can be a source of misdiagnosis. 85

In children, the most common antibodies found are anti-NMDAR, anti-MOG, and anti-GAD65. 17 86 87 88 89 90 For that reason, testing may be more focused, and if results are negative in pediatric patients with high clinical suspicion, samples should be re-evaluated in research laboratories. Among adults, despite specific clinical phenotypes and diagnostic clues, antibody positivity cannot be reliably predicted based solely on clinical presentation and ancillary investigations, 91 and testing should be performed with TBA and CBA in serum and CSF for all patients.

The data available showed that commercial tests can be limited in their accuracy; research revealed that they can yield false negative results in 14%, and CBA alone can also have false positive results in CSF. 10 92 Therefore, relying solely on isolated commercial assays can lead to misdiagnosis and diagnostic delay in some subtypes of AE, 93 and should be interpreted with caution. The current recommendation is to perform TBA to better interpret CBA.

## ENVIRONMENTAL TRIGGERS AND GENETICS

Genetics studies explored HLA associations and genome-wide association studies (GWAS) in AE. Human leukocyte antigen was linked to various AE subtypes: anti-GAD65, anti-LGI1, anti-CASPR2, anti-IgLON5, and postherpetic AE. 94 However, available data indicate that anti-NMDARE may not primarily involve HLA-related mechanisms, and exploring non-HLA regions is also crucial for understanding AE.

Anti-NMDARE has three phenotypes likely with distinct pathophysiology: postherpes simplex encephalitis, ovarian teratoma-related, and idiopathic form. Studies have identified a link between toll-like receptor 3 deficiency and postherpetic encephalitis. 95 96 In genetic analyses excluding post-herpetic cases, HLA DRB1*16:02 has been associated with the Han population, 97 in which 14% exhibited ovarian teratoma, and a weak association with HLA-B*07:02 in the German population, 98 with an 89% ovarian teratoma rate. The largest GWAS on anti-NMDAR encephalitis, involving 413 Chinese patients with a 9% rate of ovarian teratoma, demonstrated an association with HLA-DQB1*05:02 and a non-HLA region, IFIH1. 99 Further, two smaller studies have found associations with single nucleotide polymorphisms (SNPs) in non-HLA regions, such as *IRF7* , *BANK1* , and *TBX21* , 100 as well as *ACP2* , *NR1H3* , and *LRRK1* genes. 101 These results underscore the genetic variability across populations and indicate a predominant dysfunction in B cells and innate immune responses, aligning with the hypothesized disease mechanism. 102 A multiethnic study may provide deeper insights into the complex mechanisms of anti-NMDARE phenotypes.

Anti-LGI1 encephalitis is associated with *HLA-DRB1* *07:01, found in ∼ 90% of affected Caucasians and Koreans. 103 104 Notably, non-carrier patients are often younger, predominantly female, and present fewer psychiatric and frontal symptoms. 103 A multiethnic cohort with 269 patients also identified a secondary effect of DRB1*04:02 , which correlates with a lower age of onset. 104 Furthermore, a small GWAS involving 54 patients highlighted two SNPs outside the HLA region, *DCLK2* and zinc-finger genes. 98 These findings suggest potential subgroups among anti-LGI1 patients, and further research into prognostic implications is warranted.

Anti-IgLON5 encephalitis was associated with a strong HLA DQ effect, with ∼ 60% of patients carrying DQB1*05:01 ∼ DRB1*10:01 . 65 105 In a cohort of 87 patients, 85% presented DQA1*01 ∼ DQB1*05 . 106 An additional study with 35 patients revealed a significant association with MAPT H1/H1 homozygous genotype, found in 83% of patients. 105 These findings support that a primarily autoimmune process contributes to tauopathy development in genetically predisposed individuals.

A study across three phenotypes with anti-CASPR-2 ABs (LE, Morvan, and Isaacs syndromes) found an HLA DRB1*11:01 association in ∼ 90% of limbic encephalitis cases. 107 108 No association was found in the Morvan and Isaacs syndromes, suggesting distinct pathophysiological mechanisms. Interestingly, limbic encephalitis mainly involves immunoglobulin G 4 (IgG4) and is not associated with tumors, whereas Isaacs and Morvan syndromes are IgG1 predominant, and the latter is often linked with malignant thymoma. 40 108

Many familial cases of anti-GAD65 neurological syndromes, such as limbic encephalitis, ataxia, and stiff person syndrome, were reported, suggesting a genetic predisposition. 109 110 111 112 Anti-GAD was linked to DQA1*05:01 – DQB1*02:01 – DRB1*03:01 , and DQA1*03:01 – DQB1*03:02 – DRB1*04:01 , with the latter also being associated with T1DM. 101 113 Conversely, a recent GWAS with 167 patients identified 16 loci associated with anti-GAD, highlighting significant loci in the HLA class-I region and numerous genes involved in innate and adaptive immunity. 101

## TREATMENT

The initial treatment of AE included methylprednisolone (MP) plus intravenous immunoglobulin (IVIg) or plasmapheresis (PLEX). This recommendation is based on previous data showing that anti-NMDARE had better outcomes with combined initial therapy. 20 114 115 116 117

First-line treatment must be provided within < 4 weeks of symptoms onset and should not be delayed while waiting for autoantibody-testing results. The response should be monitored for 10 to 14 days after treatment initiation using clinical scales. 118 In anti-LGI1 encephalitis, initial studies advocated the efficacy of steroid monotherapy. Nonetheless, additional data suggested that anti-LGI1 patients benefit from IVIg. 32 119 120 121 The 2024 Canadian consensus, built using modified RAND methodology, ratifies this initial combined approach, especially in severe cases. 122

Rituximab (RTX) is the preferred second-line treatment for AE and should be initiated early in the disease course. The GENERATE study evaluated 358 patients diagnosed with AE and showed that RTX correlated with improved clinical outcomes and lower relapse rates, particularly in anti-NMDARE. 123 Additionally, several other reports support the efficacy of RTX in both adults and children with AE. 114 115 116 122 124

Cyclophosphamide (CYC) may be utilized as an alternative therapy if RTX is unavailable or as a second-line escalation for patients who do not respond to RTX. 114 115 124 However, it is cautioned that CYC carries a higher risk of toxicity relative to RTX and is not routinely recommended for use in children due to potential long-term adverse effects. An exception is made in severe cases, where CYC can be used for pediatric patients. 91 114

For refractory cases, third-line options such as bortezomib and tocilizumab should be considered. 123 125 126 Bortezomib may offer therapeutic benefits in patients previously treated with RTX by targeting long-lived B-cells and plasma cells in the bone marrow, although the evidence supporting its use remains limited. 124 125 A treatment protocol including tocilizumab, an interleukin-6 receptor antagonist (anti-IL6R), combined with MP, IVIg, and RTX, initiated within one month of symptom onset, has significantly shown improved functional outcomes. 127

In SPS, the recommended therapeutic approach involves GABA-enhancing medications, such as benzodiazepines (diazepam or clonazepam), baclofen, and gabapentin for symptomatic management. 128 129 Intravenous immunoglobulin is the preferred treatment, which reduces cumulative physical disability. 130 Doses begin with 2 g/kg monthly for the first 3 to 6 months, followed by adjusted dosing intervals or dosage reductions based on response. 128 130 131 Maintenance therapy continues to be effective in the long term in 67% of patients (median 3.3 years). 132 If there is no disease improvement after 3 months, RTX every 6 to 12 months may be considered. 133 The third-line treatment for SPS is autologous hematopoietic stem cell transplantation. 134 135 136 137

New perspectives in monoclonal antibody (mAb) treatments are currently under investigation, targeting long-lasting plasmablasts or plasma cells, potentially including B-cell depletion mechanisms. 29 Inebilizumab (NCT04372615) achieves a direct mechanism of action by binding to CD19 on B-cells, leading to their extensive and prolonged depletion. 42 Conversely, satralizumab (NCT05503264) achieves its effects indirectly by functioning similarly to tocilizumab, suppressing B-cell maturation. 42 Rozanolixizumab (NCT04875975) offers a novel mechanism by targeting the neonatal Fc receptor (FcRn), accelerating the degradation of unbound IgGs through lysosomal pathways. 43 Additionally, preclinical studies on chimeric autoantibody receptor T (CAR-T) therapy show promise in genetically-modifying cells to selectively target and eliminate anti-NMDARE B cells, effectively reducing autoantibody production without impacting other B-cell populations. 138 139

## CLINICAL SCALES

The mRS has been used to assess severity, response to treatment, and long-term outcomes. 16 However, the mRS emphasizes motor aspects of function independence and does not represent the variety of symptoms in AE patients. 118 The Clinical Assessment Scale in Autoimmune Encephalitis (CASE) was developed to assess the severity of the condition and to determine the response to treatment. 118 This scale includes 9 groups of symptoms and can be used both for short and long-term outcomes. 140 Both the mRS and CASE tend to improve over time but are insensible to mood, cognition, and the capacity to return to premorbid activities. 140

Cognitive outcomes must be evaluated in all patients. Screening tools, such as MMSE or Montreal Cognitive Assessment **(** MOCA), 140 and neuropsychological batteries can be used when available. Patient-reported outcome measures (PROMs) can capture other aspects of disease burden, such as quality of life and physical or emotional wellbeing. Numerous tools have already been employed, 141 but a specific tool for AE patients is still under development. 142

In conclusion, AE is a differential diagnosis of many neurological conditions, and research is rapidly advancing. General neurologists should, therefore, be familiar with the disease's clinical spectrum, clinical criteria, diagnostic testing, and potential mimics/misdiagnoses. After the diagnostic approach, it is important to assess disease severity using adequate tools (mRS, CASE) and initiate treatment promptly. These patients need long-term follow-up and screening for cognitive dysfunction, psychiatric symptoms, and capacity to return to previous activities. Increasing knowledge in this area will help mitigate long-term functional disabilities.
